# ECAMulticapa: Effectiveness of double-layered compression therapy for healing venous ulcers in primary care: a Study Protocol

**DOI:** 10.1186/s12912-016-0179-x

**Published:** 2016-10-12

**Authors:** Carmen Folguera-Álvarez, Sofia Garrido-Elustondo, José Verdú-Soriano, Diana García-García-Alcalá, Mónica Sánchez-Hernández, Oscar German Torres-de Castro, Maria Luisa Barceló-Fidalgo, Olga Martínez-González, Lidia Ardiaca-Burgués, Carmen Solano-Villarrubia, Pilar Raquel Lebracón-Cortés, Carmen Molins-Santos, Mar Fresno-Flores, Maria Carmen Cánovas-Lago, Luisa Fernanda Benito-Herranz, Maria Teresa García-Sánchez, Olga Castillo-Pla, María Sol Morcillo-San Juan, Maria Begoña Ayuso-de la Torre, Pilar Burgos-Quintana, Ana López-Torres-Escudero, Gema Ballesteros-García, Piedad García-Cabeza, Maria Ángeles de Francisco-Casado, Milagros Rico-Blázquez, ECAMulticapa Group, Raquel Perez Ballesteros, Mercedes Ruiz Medinabeitia, Angeles Sanchez Sebastian, Carmina Martinez Castaño, Maria Angeles Gallardo Ciudad, Dolores Garcia Moreno, Antonio Rodriguez Osorio, Laura Gomez Fernandez, Nuria Garcia López, Ines Casado Mora, Inmaculada Navas Cerezo, Carmen Venturini Medina, Concepción Cruz del Águilla, Isabel Jimenez Jimenez, Inmaculada Fernandez Moyano, Silvia Martinez Martinez-Treceño, Carmen Huber Alonso, Silvia Chavernas Martin, Eva Maria Luque Lora, Magdalena Garcia Paredes, Concepción Antelo Brioso, Felix Martinez Velez, Flora Espejo Matorrales, Oscar Jimenez Mercad, Cristina Casinello Espinosa, Angeles Gallego Rubio, David Villamañan Lobo, Aurora Collado Suarez, Carmen Sanchez Fuentes, Isabel Garcia Lopez, Aida Virginia Gomez Diaz, Maria Paz Barco Vega, Concepción Vela Velázquez, Luisa Mencia Vizoso, Elizabeth Rodriguez Cerón, Natividad Chasco Pérez, Esperanza Sánchez Rodriguez

**Affiliations:** 1 Centro de Salud La Paz, Atención Primaria, Servicio madrileño de Salud, Avenida Parque de Asturias s/n, Rivas-Vaciamadrid, 28523 Madrid Spain; 2 Red de investigación en Servicios de salud en enfermedades crónicas (REDISECC), Unidad de Apoyo a la Investigación, Gerencia de Atención Primaria Madrid, Calle Hacienda de Pavones, 271-28030 Madrid, Spain; 3 Facultad de Ciencias de la Salud, Departamento de Enfermería Comunitaria, Medicina Preventiva, Salud Pública e Historia de la Ciencia, Universidad de Alicante, Campus de San Vicente del Raspeig, AP 99, San Vicente del Raspeig, 03080 Alicante Spain; 4 Centro de Salud Villa de Vallecas, Atención Primaria. Servicio madrileño de Salud, Calle Fuentidueña, 12 28031 Madrid, Spain; 5 Hospital Puerta de Hierro Majadahonda, Servicio madrileño de Salud, Calle Joaquín Rodrigo 2, Majadahonda, 28222 Madrid Spain; 6 Centro de Salud Federica Montseny, Atención Primaria, Servicio madrileño de Salud, Avenida de la Albufera, 285-28038 Madrid, Spain; 7 Centro de Salud Rafael Alberti, Atención Primaria, Servicio madrileño de Salud, Calle San Claudio 154 c/v León Felipe, 28038 Madrid, Spain; 8 Centro de Salud Pavones, Atención Primaria, Servicio madrileño de Salud, Calle Hacienda de Pavones, 271-28030 Madrid, Spain; 9 Dirección Asistencial Sureste, Atención Primaria, Servicio madrileño de Salud, Avenida de la Albufera, 285-28038 Madrid, Spain; 10 Centro de Salud Paracuellos del Jarama, Atención Primaria, Servicio madrileño de Salud, Calle Chorrillo Alta, 26-28860 Paracuellos del Jarama, Madrid Spain; 11 Centro de Salud Buenos Aires, Atención Primaria, Servicio madrileño de Salud, Calle Pio Felipe, c/v Boada, c/v José Paulette, 28038 Madrid, Spain; 12 Residencia San Fernando, Consejería de Bienestar Social, Calle Ventura de Argumosa 4, 28830 San Fernando de Henares, Madrid Spain; 13 Hospital Clínico Universitario San Carlos, Calle del Prof Martín Lagos, S/N, 28040 Madrid, Spain; 14 Centro de Salud Arganda, Atención Primaria, Servicio madrileño de Salud, Calle Camino del Molino s/n, 28500 Arganda del Rey, Madrid Spain; 15 Centro de Salud San Fernando II, Atención Primaria, Servicio madrileño de Salud, Plaza de Ondarreta, 28830 San Fernando de Henares, Madrid Spain; 16 Centro de Salud Villablanca, Atención Primaria, Servicio madrileño de Salud, Calle Villablanca, 81 28032 Madrid, Spain; 17 Centro de Salud Villarejo de Salvanés, Atención Primaria, Servicio madrileño de Salud, Calle Hospital, 7-28590 Villarejo de Salvanés, Madrid Spain; 18 Centro de Salud Artilleros, Atención Primaria, Servicio madrileño de Salud, Paseo de los Artilleros s/n, 28032 Madrid, Spain; 19 Centro de Salud Mejorada del Campo, Atención Primaria, Servicio madrileño de Salud, Calle Ciudad de Paris, 22-28840 Mejorada del Campo, Madrid Spain; 20 Centro de Salud Torito, Atención Primaria, Servicio madrileño de Salud, Calle Camino de Vinateros, 140-28030 Madrid, Spain; 21 Centro de Salud Jose María Llanos, Atención Primaria, Servicio madrileño de Salud, Calle Cabo Machichaco c/v, Cabo Tarifa, 28018 Madrid Spain; 22 Centro de Salud Doctor Tamames, Atención Primaria, Servicio madrileño de Salud, Calle Alameda, 1-28820 Coslada, Madrid Spain; 23 Unidad de Apoyo a la Investigación, Gerencia Asistencial de Atención Primaria de Madrid, Madrid, Spain

**Keywords:** Venous ulcer, Compression bandage, Wound healing, Nursing, Primary health care, Quality of life

## Abstract

**Background:**

Chronic venous insufficiency, in its final stage can cause venous ulcers. Venous ulcers have a prevalence of 0.5 % to 0.8 % in the general population, and increases starting at 60 years of age. This condition often causes increased dependency in affected individuals, as well as a perceived reduced quality of life and family overload.

Local Treating chronic venous ulcers has 2 components: topically healing the ulcer and controlling the venous insufficiency. There is evidence that compressive therapy favours the healing process of venous ulcers. The studies we have found suggest that the use of multilayer bandage systems is more effective than the use of bandages with a single component, these are mostly using in Spain. Multilayer compression bandages with 2 layers are equally effective in the healing process of chronic venous ulcers as 4-layer bandages and are better tolerated and preferenced by patients. More studies are needed to specifically compare the 2-layer bandages systems in the settings where these patients are usually treated.

**Method/design:**

Randomised, controlled, parallel, multicentre clinical trial, with 12 weeks of follow-up and blind evaluation of the response variable.

The objective is to assess the efficacy of multilayer compression bandages (2 layers) compared with crepe bandages, based on the incidence of healed venous ulcers in individuals treated in primary care nursing consultations, at 12 weeks of follow-up.

The study will include 216 individuals (108 per branch) with venous ulcers treated in primary care nursing consultations.

The primary endpoint is complete healing at 12 weeks of follow-up. The secondary endpoints are the degree of healing (Resvech.2), quality of life (CCVUQ-e), adverse reactions related to the healing process. Prognosis and demographic variables are also recorder.

Effectiveness analysis using Kaplan-Meier curves, a log-rank test and a Cox regression analysis. The analysis was performed by intention to treat.

**Discussion:**

The study results can contribute to improving the care and quality of life of patients with venous ulcers, decreasing healing times and healthcare expenditure and contributing to the consistent treatment of these lesions.

**Trial registration:**

This study has been recorded in the Clinical Trials.gov site with the code NCT02364921. 17 February 2015.

## Background

### Aging and chronicity

Spain, as with other developed countries, is experiencing a progressive ageing of the population (according to the National Institute of Statistics). In 2052, the over-64 age group increased by 7.2 million, accounting for 37 % of the total population [1], contributing to an increase in chronic diseases, including chronic venous insufficiency.

In chronic venous insufficiency, the venous system of the legs is ineffective in performing venous return, and there is venous reflux due to valve failure, physical inactivity and cardiovascular problems, which lead to an increase in pressure within the veins and venous hypertension.

Chronic venous insufficiency covers a series of signs and symptoms including varicose veins, varicules, oedema, skin lesions and ulcers.

The DETECT-IVC study conducted in Spain found that 71 % of individuals in primary care consultations had some sign or symptom related to chronic venous insufficiency [2].

### Venous ulcers. Prevalence and repercussion of the problem

Venous ulcers are lesions skin loss that rest on the skin affected by stasis dermatitis, secondary to severe chronic venous insufficiency [3]. They are mainly located in the internal lateral area of the distal third of the leg. To diagnose this condition, is necessary a physical examination to confirm the presence of tibial pulses and/or a pressure gradient in the feet >60 mmHg and/or an ankle brachial index >0.8 to rule out an arterial origin [4].

Ambulatory venous hypertension represents the start of the pathophysiology of venous ulcers. The most common trigger for ulceration is trauma on the preulcerative lesions; however, the ulcers often start spontaneously on the lesions [5].

Ulcers of venous aetiology constitute between 75 and 80 % of all ulcers. They have a prevalence of 0.5 % to 0.8 % in the general population and an incidence of between 2 and 5 new cases per thousand individuals per year [4]. The female to male ratio is 3:1, and increases starting at 60 years of age [5].

The DETECT-IVC study in Spain [2] showed that 2.5 % of individuals in primary care consultations have venous ulcers. This rate increases for women and the elderly; 2 % of patients were hospitalised and 2.5 % took time off from work.

The median healing time for chronic venous ulcers varies between 75 and 90 days [6]. Half of all healed ulcers recur at 12 months of healing. Pre-existing skin disorders are a risk factor in the incidence of new relapses [5, 6].

These ulcers have abundant exudate, are often foul smelling, can get infected and cause moderate pain, which coupled with slow healing and frequent relapses affect the quality of life of patients with these ulcers [7].

### Treatment of venous ulcers. Compression bandages

Treating chronic venous ulcers requires a comprehensive approach for the patient, by treating the aetiological factors that determine their evolution, such as venous insufficiency, diet, physical inactivity and postural measures.

Local treatment has 2 components: topically healing the ulcer and controlling the venous insufficiency through the use of compressive therapy.

Evidence suggests that the wound should be treated with moist wound healing, with dressings to control the exudate and perilesional skin moisturising [8].

There is evidence that compressive therapy favours the healing process of venous ulcers. Borges et al., in a systematic review of 33 primary studies and 2 meta-analyses that included more than 1000 patients, concluded that compressive therapy increases the healing rate of venous ulcers [8]. O’Meara S et al. performed a systematic review to compare the effectiveness of using some type of compression bandages versus not using any pressure in the healing of venous ulcers and compared the effectiveness of various types of compression. The authors included 48 clinical trials with 4321 patients and concluded that compressive therapy contributes to improving the flow of venous return, decreases oedema and pain and favours the healing process of venous ulcers [9].

There are various types of compression bandages. They are classified according to the type of material (elastic/inelastic), degree of pressure (light, medium, high) and number of layers (single layer/multilayer) [10]. The light compression elastic single-layer bandages and the crepe bandage offer low pressure (15–20 mmHg for the ankle). Multilayer bandages of 2, 3 or 4 layers can combine elastic or inelastic components, reaching a pressure of 40 mmHg at the ankle.

### Advantages and disadvantages of the various types of compression

The single-layer elastic bandages with light compression maintain the necessary pressure level for 1 h, do not control the exudate and can result in overpressure when applying them and are therefore considered fixing bandages [11, 12].

Multilayer bandages are effective both at rest and in motion, they reduce the calibre of superficial and deep veins, promote venous flow, reduce oedema, improve the effect of the muscle pump of the lower legs and reduce orthostatic flow, residual volume and venous pressure by improving the operation of venous valves [13].

Various studies have compared the efficacy of the different compression therapy systems in the healing of chronic venous ulcers. O’Meara et al. performed a systematic review to compare the efficacy of 2 types of compression bandages: multilayer bandages with 4 layers and short-stretch bandage. The authors included 5 clinical trials with 797 patients. They concluded that 4-layer compression bandages are associated with a shorter healing time. The authors recommended performing more studies to compare the compression bandage systems, using a system to blind the assessment of the response variables [6].

Another study compared multilayer compression bandages with 2 and 4 layers in terms of the pressure achieved under the bandages and patient tolerance. The study found no differences in the pressure achieved by the 2 types of compression, but the 2-layer compression bandage was better tolerated by the patients [14].

Moffatt et al. compared the complete healing rate at 8 weeks of follow-up, the quality of life and the patients’ preferences when using 2 types of compression bandages (2 and 4-layer). The authors found no differences in the complete healing rate; however, the overall quality of life score and the preferences were significantly higher for patients who used the 2-layer bandages [15].

It has been estimated that treating venous ulcers generates high direct and indirect costs, increased consultations in primary care and hospitalisations. Topical and systemic treatments, represents approximately 2 % of the healthcare expenditure in Spain and neighbouring countries [2].

Published literature suggest that the use of multilayer bandage systems is more effective than the use of bandages with a single component, although the majority of the studies included 4-layer bandages. Moreover, multilayer compression bandages with 2 layers are equally effective in the healing process of chronic venous ulcers as 4-layer bandages and are better tolerated by patients who express a preference for the 2-layer bandages [15].

More studies are needed to specifically compare the 2-layer bandages systems in the settings where these patients are usually treated, using blinding techniques to assess the response variable.

In our region, light compression single-layer elastic bandages (crepe bandages) are commonly used. We are not aware of any study in Spain that has specifically compared the effectiveness of 2-layer compression bandages versus the single-layer elastic light compression bandages commonly used in health centres.

We propose this study to compare the effectiveness of 2-layer compression bandages versus crepe bandages in the healing of venous ulcers and determine how they affect the quality of life of individuals with this problem.

### Aim

The aim of this study is to assess whether multilayer compression bandages with 2 layers are more effective than crepe bandages in the healing of chronic venous ulcers in patients treated in primary care nursing consultations, measured by the incidence rate of ulcers with complete healing at 12 weeks of follow-up in each group.

The secondary objectives are to compare the effectiveness of the multilayer and crepe bandages (based on the degree of healing achieved and measured with Resvech 2.0), assess improvements in quality of life (measured with the Charing Cross Venous Ulcer Questionnaire [CCVuQ-e]) and analyse the sociodemographic, clinical and treatment factors associated with complete healing of venous ulcers.

## Methods/design

### Study design

Randomised, controlled, parallel, multicentre clinical trial, with 12 weeks of follow-up and blind evaluation of the response variable.

The study involves 22 primary healthcare centres in the Madrid region of Spain.: La Paz, Arganda, Artilleros, Buenos Aires, Federica Montseny, José Maria Llanos, Pavones, Rafael Alberti, Torito,. Villa de Vallecas, Villablanca, Villarejo de Salvanés, Mejorada del Campo, Jaime Vera, Los Alperchines, San Fernando II, Dr. Tamames, Cerro Almodovar, Ibiza, Valdebernardo, Vicente Soldevilla and Morata de Tajuña. The last six centres were added later to the study, in order to achieve the required number of patients to recruit, being approved by the Ethics Committee.

Sixty one volunteer nurses will participate in the study. The nurses will conduct the intervention.

The investigator will properly inform all study participants and will request their written, signed and dated informed consent. The investigator will provide complete and appropriate verbal and written information on the nature, purpose and potential risks and benefits of their participation in the study.

### Participants

Participants older than 18 years with chronic venous ulcers who attend to a primary care nursing consultation.

#### Inclusion criteria

- Over 18 years of age.
- Individuals with a diagnosis of venous ulcers of more than 6 weeks of evolution. If the participant has more than one lesion, the nurse will select the lesion with the highest Resvech score for the study.
- Presence of an ankle brachial index (ABI) greater than 0.8 and less than 1.3.
- Individuals who able to follow the demands of the trial and who provide their written informed consent to participate.

#### Exclusion criteria

Related to contraindications for compression therapy: Patients diagnosed with poorly controlled diabetes mellitus (latest HbA1c according to the recommendations of American Diabetes Association (ADA) and European Association for the Study of Diabetes (EASD) [16]), on treatment with antineoplastic agents, with decompensated heart failure, acute phase dermatitis, at the time of the study, rheumatoid arthritis, acute phase deep vein thrombosis, with mixed ulcers or patients who are simultaneously participating in another clinical trial.

### Sample size

Earlier studies have achieved complete healing rates at 12 weeks of follow-up of 58 % by employing multilayer bandages. For an alpha error of 0.05 and a beta error of 0.2 (80 % power) and to detect a minimum difference of 20 % between the 2 groups, a total of 97 patients will be needed in each group. Estimating a 10 % loss rate at 3 months, at least 108 patients will be included in each study branch.

The patients will be consecutively included in the study. The recruitment will be competitive until the sample size is reached.

### Random assignment

Randomisation sequence: The randomisation will be performed automatically by inserting the patients’ data into the electronic case report forms (eCRF).

Lack of knowledge of the randomisation sequence by the professional who enrols the patients will therefore be ensured.

**Fig. 1:**
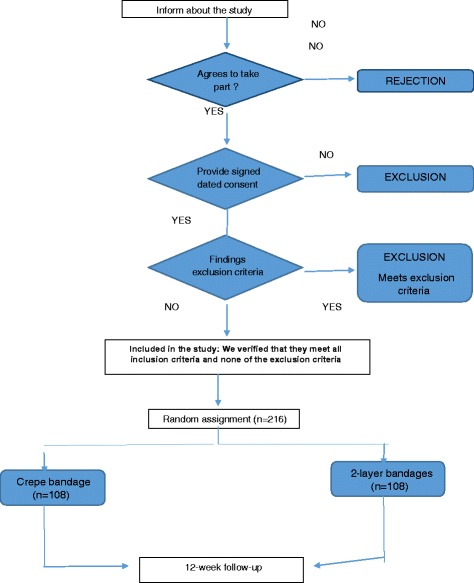
Patient Recruitment. Patients diagnosed with venous ulcers and who meet the inclusion criteria

Patient recruitment is shown in Fig. 1.

### Blinding

It is not possible to blind the intervention in this type of study. The measurement of the primary endpoint, degree of healing, will be conducted by nurses who are unaware of the type of intervention applied to the patient. The analysis will be performed by practitioners who are unaware of the assignment.

### Intervention

#### Control group

Standard clinical practice that consists of healing the wound (assessment, cleaning, disinfection, debridement and topical treatment) and application of simple compression therapy with a light compression, single-layer elastic bandage (crepe bandage).

#### Intervention group

The standard practice in healing will be conducted, modifying the application of compression therapy, which in this case will be multilayer, using 2 layers.

All patients will be provided hygienic-dietary counselling and information on adverse signs and symptoms.

Both interventions will be performed in the health centre or at home, according to the patient’s needs.

**Fig. 2:**
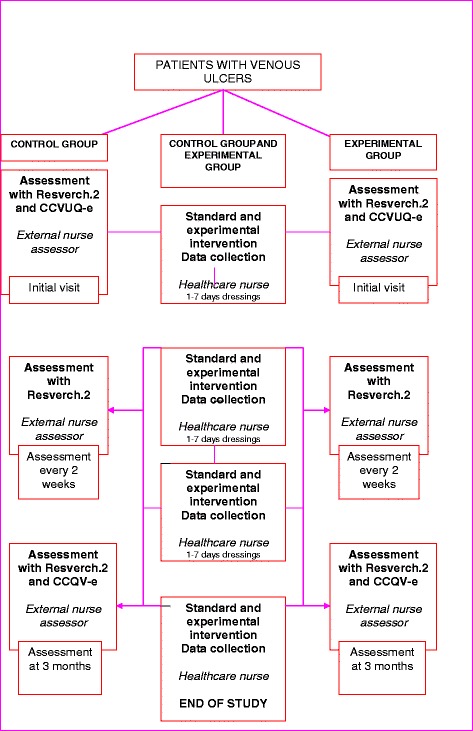
Sequence of visits in Control and Experimental Group

The sequence of visits is listed in Fig. 2.

The schedule of visits is shown in Table 1.

**Table 1:** Schedule of visits

	Baseline visit Day 0	Visit 1 Day 1	Visit 2 Day 15	Visit 3 Day 30	Visit 4 Day 45	Visit 5 Day 60	Visit 6 Day 75	Visit 7 Day 90 End of follow-up
Verification of inclusion criteria	x							
Patient has signed the informed consent	x							
Randomisation	x							
Intervention		x	x	x	x	x	x	x
Resverch assessment		x	x	x	x	x	x	x
CCVUQ-e questionnaire		x						x
Collecting data on adverse reactions			x	x	x	x	x	x
Healing complete			x	x	x	x	x	x
Sociodemographic variables	x							
Prognostic variables		x						
Variables related to healing		x	x	x	x	x	x	x

### Training

The participant nurses will receive an standardized training to minimize, as much as possible, the differences in the in the implementation of the 2 interventions. The method for performing the control technique will be consistent, and training will be provided to the nurses participating in the study on multilayer compression bandages. Both techniques will be documented in writing and supplemented with images.

To avoid differences in the assessment criteria as much as possible, prior training will be conducted for both groups. The blinded evaluators will also be trained.

### Variables

#### Outcome variables

The main outcome variable is the Complete healing at 12 weeks of follow-up: (yes/no). *complete and sustained epithelialisation for at least 2 weeks. Time elapsed between start of the study and complete healing of the wound (in days).

Secondary outcome variables are: Degree of healing (Resvech 2.0); health-related quality of life for patients with venous ulcers (CCVUQ-e) and presence of adverse reactions.

The RESVECH 2.0 scale was used to monitor wounds [17]. Based on the results of a PhD thesis, it can be used to assess characteristics and evaluate the healing process in all types of chronic wounds. It assesses six parameters: wound size, depth of the affected tissues, status of the edges, type of tissue in the wound bed, level of exudate and inflammation-infection in the wound (presence of biofilm).

The CCVUQe is a specific tool to measure quality of life with venous ulceration [7], recently validated Spanish language version of the questionnaire.

#### Sociodemographic variables

- Age, sex, living alone (yes/no), employment status and education level.

#### Variables related to the healing process

- Body mass index. Quantitative (kg/cm^2^); underlying disease; ABI; tobacco and alcohol consumption; topical and systemic treatment; patient mobility; time dedicated by patient to walking, exercise and raising the legs above the heart (minutes/day, times/week); intervention group.
- Time the patient remains with the legs above the heart (minutes/week; times/week).
- Intervention group: multilayer compression bandages with 2 layers / crepe bandages.

#### Prognostic variables

- Location of the ulcer; number of ulcers at the time of the study; time in days of evolution of the venous ulcers before inclusion in the study; recurrent ulcer (yes/no); location where the intervention is performed (home/health centre).

### Data collection

During the nursing consultation (in the health centre or at home), the individuals eligible for inclusion in the study will be informed of their eligibility and offered a chance to participate. If they accept, the patients will be checked to see if they meet all the eligibility criteria and will be requested to complete and sign the informed consent. Data collection will be performed every 14 days during the 12-week follow-up or will finish early if the ulcer has healed.

The information will be collected through clinical interviews and wound examinations, and the information will be recorded in the eCRFs. We will record those patients who decline to participate in the study (age and sex), as well as the losses, attrition and their causes. We will also record those patients who withdraw due to the withdrawal criteria.

The researchers will record the severe or unexpected adverse reactions regarding the product and will notify the principal investigator.

### Lost to follow up

Patients who fail to attend to their appointments will be contacted by telephone at least twice before they will be considered lost to follow up. The attrition rate and the reasons of ‘lost to follow up’ will be specifically collected during the study.

### Withdrawal criteria

Patients will withdraw from the study if the bandages cause them adverse reactions that preclude them from continuing the study or under circumstances that prevent them from keeping the assigned bandages or performing the follow-up visits.

### Analysis

1. Descriptive analysis of each variable with its corresponding 95 % confidence interval. Normality tests. Description of the profile of patients who withdraw from the study and their reasons for withdrawal.
2. Comparison of the group at the beginning of the trial with regard to their descriptive and response variables and predictors. Bivariate statistical tests will be employed that are suitable for the variable type (qualitative or quantitative).
3. Primary effectiveness analysis: A comparison will be performed of the incidence rates of ulcers with complete healing, using the ratio of incidence rates, with their point estimate and 95 % confidence interval. We will compare the time to complete healing using Kaplan-Meier curves for both types of treatment (log-rank test). To adjust for prognostic factors, we will perform a Cox regression model, with the time elapsed to complete healing as the dependent variable, complete healing as the event and the intervention group as the independent variable. We will include in the model those variables that can act as confounding factors or that can modify the effect.
4. Secondary effectiveness analysis: For each secondary variable, we will apply the appropriate statistical technique based on the character of each variable (qualitative or quantitative) and to the distribution to which they fit (parametric or nonparametric).

The individuals responsible for performing the analysis will be unaware of which intervention each patient included in the study has undergone. All statistical analyses will be performed by intent-to-treat, using the conventional level of statistical significance (0.05).

## Discussion

The aim of our study is to measure the effectiveness of an intervention for treating chronic venous ulcers in the setting of a primary care nursing consultation. Chronic venous ulcers are a prevalent condition that mainly affects populations older than 65 years of age. Patients affected, due to their age and the presence of concomitant diseases, are considered an especially vulnerable population from a healthcare point of view. This pragmatically designed clinical trial, conducted in a primary care setting and that evaluates various nursing interventions, is ultimately aimed at improving the management of patients with venous ulcers. If the multilayer compression bandages with 2 layers show greater effectiveness in healing venous ulcers than the crepe bandages employed in standard practice, the former’s application in clinical practice will result in better and quicker healing of these wounds. This in turn will result in reduced treatment costs, optimising the human and material resources of the healthcare system. Moreover, it will contribute to standardising the intervention for these patients, thereby promoting equal treatment.

Given the nature of the intervention, it is impossible to blind it. However, we have designed a blind assessment by nurses who are unaware of the patient assignment, and the analysis will be performed by professionals who are also unaware of the assignment.

The patients will be recruited and treated by their own reference nurses. Although this approach may increase the variability of the intervention technique, we will try to minimize it through the prior training to all participant nurses. We have also designed a specific eCRFs for data collection and al the procedures have been standardized.

Although there might be variability in the use of healthcare products used for healing and therapeutic adjustment, this variability is minimal, given that the Primary Care Management implemented an Improvement Plan in 2010 for the Care of Patients with Chronic Skin Ulcers. The plan provided specific training for professionals, a design for Recommendations guidelines [3] and a centralised procurement procedure. The availability of these products is therefore standardised and regulated for all health centres.

## Abbreviations

ABI: Ankle brachial index
AHT: Arterial hypertension
CCVuQ-e: Charing cross venous ulcer questionnaire
COPD: Chronic obstructive pulmonary disease
DVT: Deep vein thrombosis
EC: Clinical research ethics committee
eCRF: Electronic case report forms
IC: Informed consent
NSAIDS: Nonsteroidal anti-inflammatory drugs

## References

[1] Long-term population projections in Spain 2012–2052. Available at http://www.ine.es. Accessed 23 May 2015.

[2] Alvarez-Fernández LJ, Lozano F, Marinel lo-Roura J, Masegosa-Medina JC (2008). Encuesta epidemiológica sobre la insuficiencia venosa crónica en España: estudio DETECT-IVC 2006. Angiologia.

[3] Servicio Madrileño de Salud. Recomendaciones para el tratamiento local de las úlceras cutáneas crónicas de la Comunidad de Madrid. Madrid: Comunidad de Madrid. Consejería de Sanidad. 2010. ISBN-84: 978-84-690-7802-0.

[4] Conferencia Nacional de Consenso sobre Úlceras de la Extremidad Inferior. Documento de Consenso CONUEI. Barcelona: CONUEI; 2008.

[5] Marinel-lo J. Úlceras de la extremidad inferior. Barcelona. Editorial Glosa; 2005

[6] O’Meara S, Tierney J, Cullum N, Martin J, Frank P, Nule T (2009). Four layer bandage compared with short stretch bandage for venous legs ulcers: systematic review and metaanalysis of randomised controlled trial with data from individual patients. BMJ..

[7] González Consuegra RV (2011). Calidad de vida y cicatrización en pacientes con úlceras de etiología venosa: adaptación transcultural y validación del “Charing Cross Venous Ulcer Questionnaire (CCVUQ)” y del “Pressure Ulcer Scale for Ealing (PUSH)” [tesis doctoral].

[8] Borges Eline Lima, Caliri Maria Helena Larcher, Haas Vanderlei José. Revisión sistemática del tratamiento tópico de la úlcera venosa. Rev. Latino-Am. Enfermagem [Online newspaper]. 2007. Dez ;15:1163–1170. Available at http://www.scielo.br/scielo.php?script=sci_arttext&pid=S0104-11692007000600017&Ing=en. Accessed 11 April 2011. doi:10.1590/S0104-11692007000600017 10.1590/s0104-11692007000600017 18235960

[9] O’Meara S, Cullum S, Nelson E. Compresión para las úlceras venosas de las piernas (Cochrane traducida) En: Biblioteca Cochrane Plus 2009 Número 2. Oxford: Update Software Ltd O’Meara S, Cullum N, Nelson EA,Dumville JC. Compression for venous leg ulcers. Cochrane Database Syst Rev. 2012;(11):CD000265. doi:10.1002/14651858.CD000265.pub. 10.1002/14651858.CD000265.pub3 PMC7068175 23152202

[10] Asociación Profesional de Enfermeras de Ontario (Registered Nurses Association of Ontario, 2007) (2007). Valoración y manejo de las úlceras venosas en la pierna.

[11] Colegio de Enfermería de Teruel. Heridas crónicas. Available at http://www.enferteruel.com/. Accessed 5 May 2012.

[12] Tavizon Ramos OE, Alanzón-Romero P (2009). Algunos aspectos clínicos y patológicos de la úlcera de pierna. Dermatolg Rev Mex.

[13] Torra i Bou JE, Rueda López J, Blanco Blanco J, Torres Ballester J, Toda LL (2003). Úlceras venosas ¿Sistema de compresión multicapa o venda de crepe?. Rev ROL Enf.

[14] Junger M, Ladwing A, Bohbot T, Haase H (2009). Comparison of interface pressures of the compression bandaging systems used on healthy volunteers. J Wound Care.

[15] Moffatt J, Edwuard C, Collier M, Treadwell T, Miller M, Shafe T (2008). A randomised controlled 8 week crossover clinical evaluation of the · M Coban 2 layer compression system versus Profore to evaluate the product performance to patients with venous leg ulcers. Int Wound J.

[16] Inzucchi SE, Bergenstal RM, Buse JB, Diamant M, Ferrannini E, Nauck M (2012). American Diabetes Association (ADA); European Association for the Study of Diabetes (EASD). Management of hyperglycemia in type 2 diabetes: a patient-centered approach: position statement of the American Diabetes Association (ADA) and the European Association for the Study of Diabetes (EASD). Diabetes Care.

[17] Restrepo Medrano, JC. Verdú Soriano, J. Instrumentos de monitorización clínica y medida de la cicatrización en úlceras por presión (UPP) y úlceras de la extremidad inferior (UEI). Desarrollo y validación de un índice de medida, 2010. Doctoral thesis [online]. http://gneaupp.info/instrumentos-de-monitorizacion-clinica-y-medida-de-la-cicatrizacion-en-ulceras-por-presion-y-ulceras-de-la-extremidad-inferior-desarrollo-y-validacion-de-un-indice-de-medida/. Accessed 23 Jan 2012.

